# Effect of different levels of *Scutellaria baicalensis* straw on slaughter performance and rumen microorganisms of rams

**DOI:** 10.1371/journal.pone.0325708

**Published:** 2025-06-10

**Authors:** Minghui Zhang, Liangzhong Hou, Yuxia Yang, Hua Zhang, Zhijun Zhang, Jinlong Li, Congbin Xu, Tongjun Guo

**Affiliations:** 1 Institute of Feed Research, Xinjiang Academy of Animal Science, Urumqi, China; 2 College of Life Science, Xinjiang Agricultural University, Urumqi, China; 3 Xinjiang Key Laboratory of Herbivorous Livestock Feed Biotechnology, Xinjiang Academy of Animal Science, Urumqi, China; Tokat Gaziosmanpaşa University: Tokat Gaziosmanpasa Universitesi, TÜRKIYE

## Abstract

The shortage of forage supply has become an issue for the development of high-quality animal husbandry. Utilizing functional diets can mitigate forage shortages while enhancing animal performance and product quality. This study aimed to investigate the effects of different levels of *Scutellaria baicalensis* straw (SBS) on the slaughter performance, rumen fermentation, and microbial diversity of rams. A single-factor completely randomized experimental design was adopted. Sixty 4- to 5-month-old Bainuburke rams with mean body weight of (27.08 ± 3.12) kg were randomly divided into six groups, with 10 per group. Each group received a balanced total mixed pellet diet with equivalent energy and nitrogen levels containing 0%, 6%, 12%, 18%, 24%, and 30% SBS (dry matter basis). The pre-experimental period was 10 days, followed by a 70 days formal experimental period. The results showed that: (1) Compared with the CON group, the dressing percentage and eye muscle area in the SBS24 group significantly increased (*P* < 0.05); (2) Compared with the CON group, the butyrate content and total fatty acid content inthe SBS24 group significantly increased (*P* < 0.05); (3) Compared with the CON group, the Simpson index of the SBS groups significantly increased (*P* < 0.05), while the Shannon index of the SBS12, SBS18, and SBS24 groups significantly decreased (*P* < 0.01). At the phylum level, the relative abundance of Firmicutes, Bacteroidetes, and Halobacteria increased, while the relative abundance of Proteobacteria and Actinobacteria decreased. At the genus level, the relative abundance of *Prevotella*, *Rikenellaceae*_RC9_gut_group, and *Succiniclasticum* increased. In summary, diets containing 6–30% SBS increased the relative abundance of Firmicutes, Bacteroidetes, and Halobacteria, while decreasing the relative abundance of Proteobacteria and Actinobacteria. This led to higher total volatile fatty acids, which in turn improved the slaughter performance of rams. Based on performance and microbiota composition, it is recommended that rams diets be supplemented with 24% SBS.

## 1 Introduction

With the rapid development of the traditional Chinese medicine (TCM) industry, the scope and scale of TCM cultivation have been continuously expanding. In recent years, interest in TCM has increased significantly, and many TCMs or their by-products have been found to be suitable as feed additives for livestock and poultry, with the added advantage that they are free of pollutants and contaminants [1]. However, most by-products of TCM are burned or discarded, leading not only to waste of resources but also to environmental pollution [2]. Therefore, the development of new, safe, and stable TCM resources is of great significance for the efficient development of animal husbandry.

*Scutellaria baicalensis* (Baikal skullcap), a perennial herbaceous plant belonging to the genus *Scutellaria* in the family Lamiaceae [3], possesses various biological functions, including antioxidant, antipyretic, analgesic, anti-inflammatory, antiallergic, antibacterial, immunomodulatory, and anticancer effects [4–5]. While its roots are used for medicinal purposes, the straw part is usually discarded, leading to resource wastage and environmental contamination [6]. The annual production of SBS in China is approximately 63,000 tons, making it a relatively abundant resource. Moreover, SBS has a crude protein (CP) content of 6.08–7.36%, higher than that of corn stover and wheat straw, making it a promising feed alternative [7]. Additionally, the main chemical components of SBS are similar to those of its roots and are rich in various flavonoid compounds [8], which have antibacterial, anti-inflammatory, antipyretic, analgesic, and antioxidant effects [9], showing potential for development as a functional forage resource.

Studies have shown that adding *S. baicalensis* to the diet of weaned piglets can alter the composition of the gut microbiota, with certain bacterial taxa such as the Prevotellaceae_NK3B31 group and Prevotella_1 showing a negative correlation with the apparent digestibility of EE [10]. Wei et al. [11] found that adding 10 g/(head·d) of a TCM additive mainly composed of *S. baicalensis* could increase the rumen pH of sika deer during the antler-growth period, enhance the abundance of Firmicutes and the genus *Rikenellaceae*_RC9 in the rumen, and promote the degradation of fibrous materials and carbohydrates. It also reduced the abundance of Actinobacteria and *Pseudomonas*, which is beneficial for animal health. Zhang et al. [12] demonstrated that adding 6%−9% SBS straw to the diet could improve the antioxidant capacity of cattle, significantly reduce the ratio of Firmicutes to Bacteroidetes, and decrease harmful intestinal bacterial populations and inflammatory responses, thereby promoting the healthy growth of beef cattle.

Currently, most research on *S. baicalensis* focused on its extracts, while its straw, as a by-product, has been rarely studied as a supplement for animal husbandry. The effects of SBS on the slaughter performance, rumen fermentation, and microbial community of ruminants are largely unknown. We hypothesized that increasing SBS inclusion will enhance fermentation efficiency and improve slaughter performance up to an optimal level, providing a scientific basis and reference for its practical application in ruminant production.

## 2 Materials and methods

### 2.1 Ethics committee approval

All animal care and handling procedures in this study were conducted in accordance with the “Guidelines for the Care and Use of Laboratory Animals in China” and were approved by the Institutional Animal Care and Use Committee of the Xinjiang Academy of Animal Science, with the identification number NO. 5 20231228 and approval date of September 13, 2023. To minimize stress during handling, transport, and slaughter, animals were maintained in a quiet environment, handled calmly by trained personnel, and transported under humane conditions. Slaughter was conducted using methods designed to ensure minimal distress and rapid processing.

### 2.2 Experimental materials

**Fig 1:**
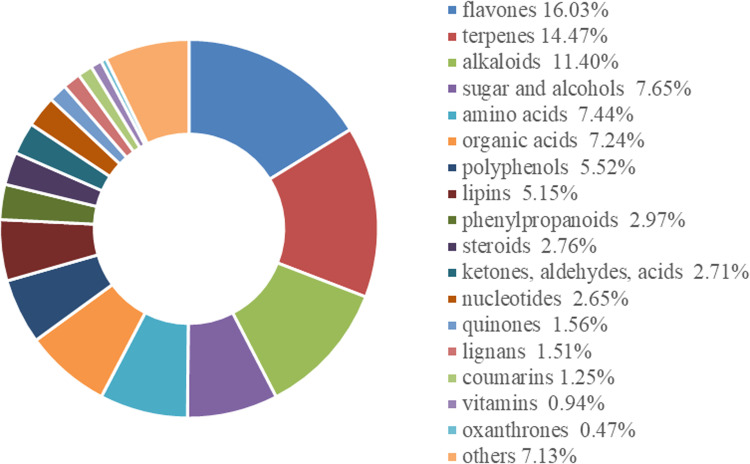
Classification of bioactive substances in SBS.

SBS was a by-product consisting of air-dried stems and leaves remaining after the roots of *S. baicalensis* were harvested in the autumn of 2022. It was provided by the Alimu Breeding Cooperative in Heshuo County, Bayingol Mongolian Autonomous Prefecture, Xinjiang, China. The nutrient composition of the SBS is shown in Table 1. Samples of SBS were sent to Biomarker Technologies Co., Ltd., Beijing, for extensive metabolite analysis using liquid chromatography–mass spectrometry (LC-MS). Fig 1 shows the categories of bioactive substances in SBS. A total of 1,921 bioactive compounds were detected and categorized into 18 types. Among them, flavones accounted for the highest proportion (16.03%), followed by terpenes (14.47%), alkaloids (11.40%), sugars and alcohols (7.65%), amino acids (7.44%), and organic acids (7.24%).

**Table 1:** Nutritional composition of SBS (DM basis, %).

Items	Nutritional content
Dry matter (DM)	96.54
Crude protein (CP)	6.30
Ether extract (EE)	2.12
Neutral detergent fiber (NDF)	60.82
Acid detergent fiber (ADF)	42.54
Crude ash (Ash)	7.88
Calcium (Ca)	0.79
Phosphorus (P)	0.17

### 2.3 Experimental design, diets, and management

The study was conducted at the Alimu Breeding Cooperative Sheep Farm in Heshuo County, Bayingol Mongolian Autonomous Prefecture, Xinjiang, China. A total of 60 Bainuburke rams, aged 4–5 months and with a body weight of (27.08 ± 3.12) kg, were randomly divided into six groups of 10 animals each using randomization software: (1) control, CON, (2) 6% SBS, (3) 12% SBS, (4) 18% SBS, (5) 24% SBS, and (6) 30% SBS. Each group of 10 replicates, were housed together in a single pen, with feed administered collectively. Each group was fed a total mixed pellet diet with equal energy and nitrogen content containing the different percentages of SBS on a dry matter (DM) basis). The experimental period was 80 days, with a pre-experimental acclimation period of 10 days and a formal experimental period of 70 days. The experimental diets were formulated according to the “Nutrient Requirements of Meat Sheep” (NY/T816-2021), containing equal energy and nitrogen, to meet the requirements of fattening sheep with a body weight of 30 kg and a daily weight gain of 200 g. The roughage was crushed through a 20 mm sieve and mixed evenly into the feeds, then processed into cylindrical pellets with a diameter of 8 mm using an SZLH-420 pellet mill (Hengfu, Xinxiang, China). During the pelletization process, the moisture content was maintained at 15%, and the feed was stored under ambient temperature conditions. The composition and nutrient levels of the experimental diets were verified by laboratory analysis and reference to established feed composition tables (Table 2).

**Table 2:** Composition and nutrient levels of the diet (DM basis, %).

Items	Control	SBS6	SBS12	SBS18	SBS24	SBS30
Ingredients						
Alfalfa hay	14.73	13.42	12.34	10.88	9.47	7.82
Wheat stalk	25.28	20.57	15.66	11.11	6.54	2.18
SBS	0.00	6.00	12.00	18.00	24.00	30.00
Corn	33.74	33.73	33.83	33.74	33.73	33.72
Wheat bran	7.18	7.19	7.17	7.23	7.18	7.03
Cottonseed meal	14.96	14.96	14.88	14.92	14.97	15.14
NaCl	1.00	1.00	1.00	1.00	1.00	1.00
NaHCO3	1.61	1.61	1.61	1.61	1.61	1.61
Premix	1.50	1.50	1.50	1.50	1.50	1.50
Total	100.00	100.00	100.00	100.00	100.00	100.00
Nutritional level						
ME/(MJ/kg)	9.20	9.30	9.20	9.22	9.22	9.16
CP	13.34	13.42	13.34	13.38	13.36	13.34
EE	2.36	2.42	2.48	2.54	2.60	2.65
NDF	44.82	43.88	44.76	44.60	44.59	45.17
ADF	27.49	27.45	27.30	26.24	27.16	28.53
Ca	1.27	1.38	1.49	1.53	1.51	1.59
P	0.38	0.42	0.38	0.36	0.39	0.40

Notes: One kg of premix contained the following: VitA 150,000 IU, VitD3 56,500 IU, VitE 8000 IU, Se (sodium selenite)14 mg, I (potassium iodide) 80 mg, Cu (copper sulfate) 290 mg, Mn (manganese sulfate) 1925 mg, Zn (zinc oxide) 2050 mg, and Co (cobalt sulfate) 24 mg. The amount of digestible crude protein in the feed was calculated by the ME given in the attached list of nutrient requirements of meat-type sheep and goats: (NY/T 816–2021); the other indicators were measured values.

Before the start of the experiment, the sheep pens were disinfected and sterilized. All experimental rams were dewormed orally with Ivermectin before the experiment and were marked with ear tags. The rams were group-housed in the same barn and fed at 09:00 and 18:00 daily. The experimental period was 80 days (2023.09–2023.12), During the experiment, the rams had free access to feed and water. Each group was ensured to have 10% residual feed and the pens were maintained in a dry and well-ventilated condition.

### 2.4 Sample collection and measurements

#### 2.4.1 Slaughter performance.

At the end of the experiment, the rams were fasted for 12 hours and deprived of water for 2 hours. Five rams from each group were randomly selected, and their live weight before slaughter (kg) was measured and recorded without any correction factors applied. The rams were then electrically stunned and exsanguinated through the jugular vein. After removing the head, hooves, skin, and viscera (while retaining the kidneys), the hot carcass weight (HCW) was measured and recorded after 30 minutes. The dressing percentage (%) was calculated using the formula:

Dressing Percentage (%) = HCW/live weight before slaughter in kg × 100%

The longissimus muscle between the 12th and 13th ribs was cut, and the cross-sectional area of the eye muscle was outlined on sulfuric acid paper. The longest and widest dimensions (cm) were measured using a Vernier caliper. The eye muscle area (cm²) was calculated using the formula [13]:

Eye Muscle Area (cm^2^) = longest dimension (cm) × widest dimension (cm) × 0.7

#### 2.4.2 Rumen fermentation parameters and microbiota.

After slaughter, the rumen contents were immediately collected from the upper left side of the rumen. The rumen fluid was filtered through four layers of cheesecloth and the first 50 mL was discarded to avoid contamination, retaining 30 mL. The pH of the rumen fluid was measured in triplicate immediately after slaughter using a portable pH meter (PHS-3C, Shanghai, China). The remaining samples were transferred into 15 mL and 5 mL cryovials and stored at −20 °C and −80 °C, respectively. The concentration of ammonia nitrogen was determined using the phenol-hypochlorite colorimetric method [14], and the concentrations of volatile fatty acids (acetate, propionate, butyrate, isobutyrate, valerate, and isovalerate) were measured by gas chromatography [15].

#### 2.4.3 DNA extraction and 16S rDNA sequencing.

The TGuide S96 DNA kit (Tiangen, Beijing, China) was used for DNA extraction according to the manufacturer’s instructions. The concentration of the extracted DNA was measured using the Qubit dsDNA HS assay kit and Qubit 4.0 fluorometer (Invitrogen, Thermo Fisher Scientific, Oregon, USA). DNA integrity was assessed by 1% agarose gel electrophoresis.

The universal primers 338F (5’-ACTCCTACGGGAGGCAGCA-3’) and 806R (5’-GGACTACHVGGGTWTCTAAT-3’) were used to amplify the V3-V4 region of the 16S rRNA gene in the extracted genomic DNA of each sample. Both the forward and reverse 16S primers were tagged with Illumina index sequences specific to each sample. The total volume of the PCR reaction was 20 μL, formulated according to Table 3, and the PCR cycling conditions were as follows: initial denaturation at 95°C for 5 minutes, followed by 25 cycles of denaturation at 95°C for 30 seconds, annealing at 50°C for 30 seconds, and extension at 72°C for 40 seconds, with a final extension at 72°C for 7 minutes.

**Table 3:** PCR reaction system.

Items	Sample/20 μL
DNA	1 μL
forward primer (10 μM)	0.3 μL
reverse primer (10 μM)	0.3 μL
KOD FX Neo Buffer	5 μL
dNTP (2 mM each)	2 μL
KOD FX Neo	0.2 μL
ddH2O	11.2 μL

The PCR amplicons were purified using Agencourt AMPure XP beads (Beckman Coulter, Indianapolis, IN) and quantified using the Qubit dsDNA HS assay kit and Qubit 4.0 fluorometer (Invitrogen, Thermo Fisher Scientific, Oregon, USA). After individual quantification, the amplicons were pooled in equimolar amounts. The constructed library was sequenced using the Illumina Novaseq 6000 platform (Illumina, San Diego, CA, USA).

### 2.5 Statistical analysis

Statistical analyses were performed using the ANOVA function in SPSS 26.0 software to conduct one-way analysis of variance. Duncan’s multiple range test was used for post hoc comparisons. Results are presented as mean ± SEM. *P* < 0.05 for significant differences and *P* < 0.01 for highly significant differences.

For microbial community analysis, the SILVA database (version 138) was used with a Bayesian classifier to classify representative sequences and annotate operational taxonomic units (OTUs). Alpha diversity was assessed using ACE, Chao1, Simpson, and Shannon indices to evaluate species richness and diversity. Beta diversity was analyzed using principal coordinate analysis (PCoA) and non-metric multidimensional scaling (NMDS) to assess differences among groups. Linear discriminant analysis effect size (LEfSe) was used to compare differences among groups.

## 3 Results and analysis

### 3.1 Slaughter performance

As shown in Table 4, the dressing percentage increased with increasing % SBS supplementation, peaking at 24% (*P* < 0.05). The eye muscle area of the SBS24 group was significantly higher than that of the CON (*P* < 0.01) and higher than that of the SBS30 group (*P* < 0.05), with a linear increase as the level of SBS increased (*P *= 0.001). However, there were no significant differences in live weight before slaughter and carcass weight between the SBS groups and the CON group (*P* > 0.05).

**Table 4:** Effect of SBS on slaughter performance (dressing %) of rams (n = 5).

Items	Group						SEM	*P*-value		
	CON	SBS6	SBS12	SBS18	SBS24	SBS30		Trt	L	Q
Live Weight Before Slaughter(kg)	37.88	40.30	40.82	39.86	39.84	38.70	0.597	0.773	0.892	0.168
Carcass Weight(kg)	18.00	20.54	20.14	20.52	20.88	19.96	0.408	0.393	0.190	0.130
Dressing Percentage(%)	47.47^b^	50.92^ab^	49.52^ab^	51.45^ab^	52.38^a^	51.32^ab^	0.563	0.024	0.026	0.274
Eye Muscle Area(cm2)	18.06^Bc^	18.65^ABbc^	19.05^ABbc^	21.51^ABab^	22.38^Aa^	20.91^ABbc^	0.453	0.006	0.001	0.328

Notes: Trt = treatment effect, L = linear, Q = quadratic; different letters (A,B,a,b,c) indicate significant (*P* < 0.05) and extremely significant (*P* < 0.01) differences within a row; SEM is the pooled standard error between six groups.

### 3.2 Rumen fermentation parameters

As shown in Table 5, there were no significant differences in rumen pH and NH_3_-N concentration among the groups (*P* > 0.05). The concentration of acetate exhibited a quadratic change with an initial increase and then a decrease as the level of SBS increased, reaching a peak at SBS12 (*P* = 0.043). Compared with the CON group, the butyrate concentration and TVFA in the SBS24 group significantly increased (*P* < 0.05), with a linear increase in butyrate concentration (*P* = 0.025) and a quadratic change in TVFA (*P* = 0.029). Specifically, the butyrate concentration in the SBS24 group increased by 48% over the CON group, and TVFA increased by 19% over the CON group.

**Table 5:** Effect of SBS on rumen fermentation (n = 5).

Items	Groups						SEM	*P*-value		
	CON	SBS6	SBS12	SBS18	SBS24	SBS30		Trt	L	Q
pH	7.43	7.43	7.37	7.40	7.31	7.39	0.034	0.921	0.466	0.684
NH3-N (mol/dL)	23.54	23.01	23.02	22.85	22.00	23.75	0.931	0.078	0.884	0.848
Acetate (mmol/L)	59.41	63.61	66.24	68.50	71.38	60.07	1.660	0.580	0.387	0.043
Propionate (mmol/L)	17.34	18.73	18.82	19.15	19.40	18.97	0.494	0.898	0.340	0.480
Isobutyrate (mmol/L)	0.39	0.43	0.42	0.44	0.43	0.42	0.016	0.977	0.729	0.489
Butyrate (mmol/L)	2.67^b^	3.34^ab^	3.71^ab^	3.76^ab^	3.94^a^	3.70^ab^	0.151	0.030	0.025	0.108
Isovalerate (mmol/L)	0.52	0.52	0.58	0.59	0.60	0.58	0.012	0.217	0.322	0.284
valerate (mmol/L)	0.23	0.23	0.22	0.24	0.25	0.25	0.011	0.982	0.543	0.868
TVFA (mmol/L)	80.57^b^	86.87 ^ab^	90.03 ^ab^	92.67 ^ab^	96.00^a^	83.98 ^ab^	1.857	0.032	0.200	0.029
Acetate/Propionate	3.46	3.72	3.37	3.58	3.81	3.27	0.102	0.843	0.922	0.596

NH3-N = ammonia nitrogen; TVFA = total volatile fatty acids.

### 3.3 Rumen bacterial abundance, diversity, and composition

#### 3.3.1 OTU clustering analysis.

**Fig 2:**
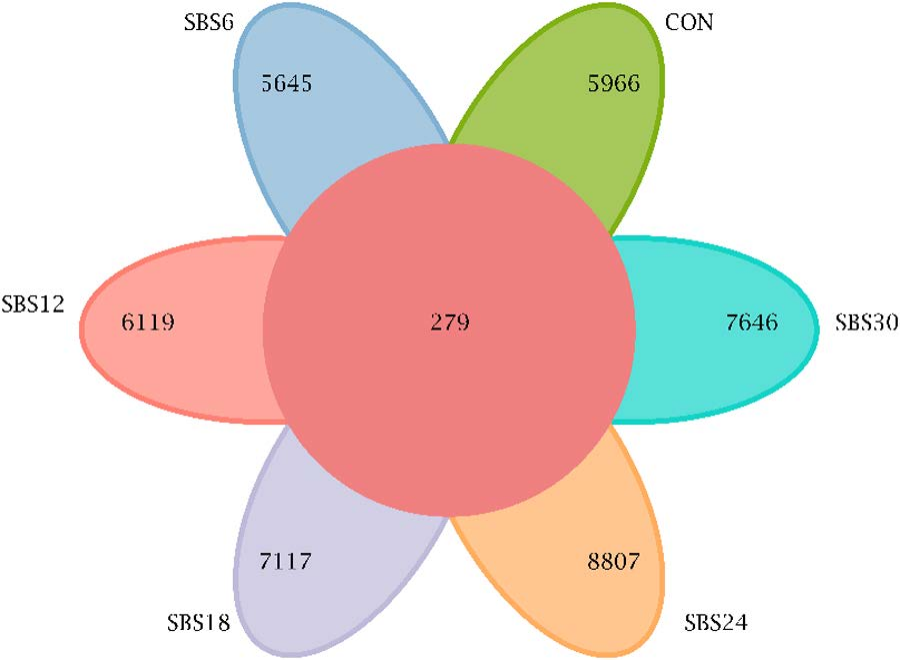
OTU venn diagram.

A total of 2,395,120 paired-end reads were obtained from the sequencing of 30 samples, which were quality-controlled and merged into 2,194,116 clean reads. Each sample produced at least 69,790 clean reads, with an average of 73,137 clean reads per sample. At a 97% similarity threshold, 45,400 OTUs were identified across all samples (Fig 2). The SBS24 group had the highest number of unique OTUs (8,807), compared to 5,966 in the CON group, 5,645 in the SBS6 group, 6,119 in the SBS12 group, 7,117 in the SBS18 group, and 7,646 in the SBS30 group.

#### 3.3.2 Alpha diversity analysis.

As shown in Table 6, the coverage values were close to 1, indicating that the sequencing results adequately reflected the rumen microbial community structure. The Simpson index was significantly higher in the SBS groups compared to the CON (*P* < 0.05). The Shannon index was significantly lower in the SBS12, SBS18, and SBS24 groups compared to the CON (*P* < 0.01), and it was also significantly lower in the SBS24 group compared to the SBS30 group (*P* < 0.01), with a quadratic change observed as the level of SBS increased (*P *= 0.001). No significant differences were observed in OTU numbers, ACE index, or Chao1 index among the groups (*P* > 0.05).

**Table 6:** Effects of different levels (%) of SBS in the diets on rumen microbial α-diversity (n = 5).

Items	Group						SEM	P-value		
	CON	SBS6	SBS12	SBS18	SBS24	SBS30		Trt	L	Q
OUT number	1589.60	1714.00	1834.80	2080.60	2360.60	2126.20	121.262	0.479	0.063	0.676
ACE index	1599.11	1729.40	1851.14	2098.19	2374.31	2139.83	121.665	0.479	0.063	0.665
Chao1 index	1590.36	1715.77	1836.58	2082.31	2361.50	2127.40	121.244	0.479	0.063	0.675
Simpson index	0.98^b^	0.99^a^	0.99^a^	0.99^a^	0.99^a^	0.99^a^	0.001	0.025	0.109	0.307
Shannon index	9.50^Aa^	8.65^BCbc^	8.34^BCc^	8.42^BCc^	8.29^Cc^	8.97^ABb^	0.098	0.001	0.111	0.001
Coverage	1.00	1.00	1.00	1.00	1.00	1.00	0.001	0.541	0.419	0.118

#### 3.3.3 Beta diversity analysis.

**Fig 3:**
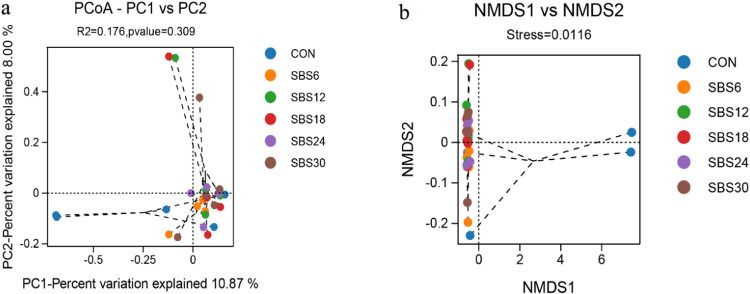
Beta diversity analysis of rumen flora. (a) PCoA principal axis analysis; (b) NMDS, non-metric multidimensional scaling analysis.

Principal coordinate analysis (PCoA) (Fig 3a) and non-metric multidimensional scaling (NMDS) (Fig 3b) based on the Bray-Curtis distance matrix showed no clear separation among the rumen microbial communities of different treatment groups, indicating that the addition of SBS did not significantly affect the beta diversity of rumen microbiota.

### 3.3.4 Microbial community structure

**Fig 4:**
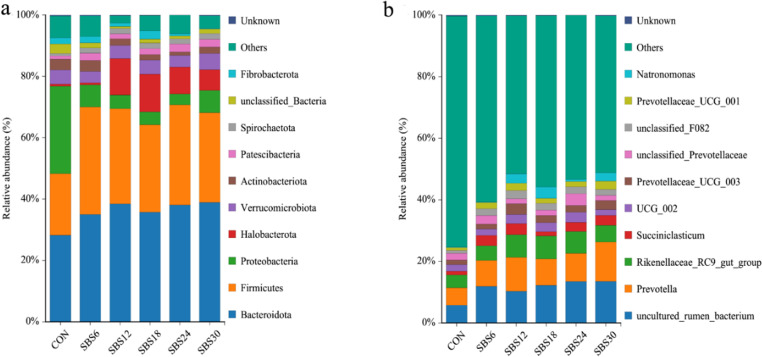
Distribution of bacterial taxa as phyla (a) and genera (b) across the different treatment groups (as a percentage of the total).

At the phylum level, Firmicutes increased in all SBS groups, while Proteobacteria decreased compared with CON (Fig 4a). The relative abundance of Firmicutes, Bacteroidetes, and Halobacteria increased in the SBS groups compared to the CON group, while the relative abundance of Proteobacteria and Actinobacteria decreased. At the genus level, *Prevotella*, *Rikenellaceae*_RC9_gut_group, and *Succiniclasticum* were dominant in both the SBS and CON groups (Fig 4b). The relative abundance of these genera increased in the SBS groups compared to the CON group.

### 3.3.5 LEfSe analysis

**Fig 5:**
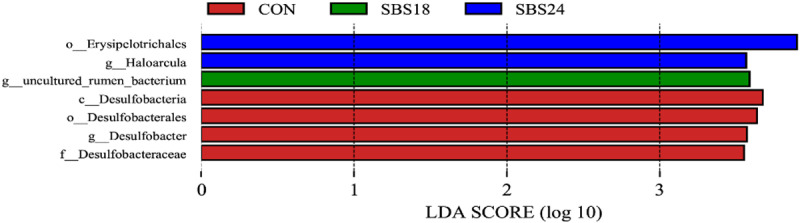
Histograms of LDA scores calculated for each taxon from phylum to genus.

As shown in Fig 5, seven taxa with significant differences in abundance were identified among the groups with LDA scores >3.0. The CON group was enriched in Desulfobacterales, Desulfobacteraceae, and *Desulfobacter* at the order, family, and genus levels, respectively. The SBS18 group was enriched in uncultured_rumen_bacteria at the genus level, while the SBS24 group was enriched in Erysipelotrichales at the order level and *Haloarcula* at the genus level.

### 3.3.6 Correlation of microbial communities with rumen fermentation parameters and slaughter performance

**Fig 6:**
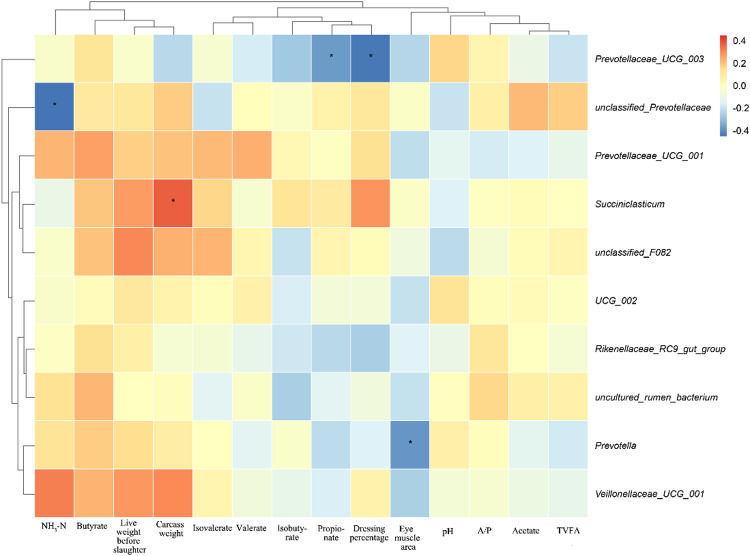
Rumen fermentation parameters and slaughter performance compared with bacterial prevalence at the genus level. Different colors represent positive correlation (red) or negative correlation (blue), and the color shade indicates the magnitude of the correlation, * 0.01 < *P* ≤ 0.05 and **P ≤ 0.01.

The heatmap of correlation between bacterial taxa in the rumen and rumen fermentation parameters and slaughter performance is shown in Fig 6. Carcass weight was positively correlated with Succiniclasticum (*P* < 0.05), eye muscle area was negatively correlated with Prevotella (*P* < 0.05), slaughter rate and propionic acid were negatively correlated with Prevotellaceae_UCG_003 were negatively correlated (*P* < 0.05), NH3-N was negatively correlated (*P* < 0.05) with unclassified_Prevotellaceae.

## 4 Discussion

### Effects of Scutellaria baicalensis straw (SBS) on ram slaughter performance

Few studies have investigated the effects of SBS on the slaughter performance of rams. However, several studies have shown that plants or extracts rich in flavonoids can improve animal slaughter performance. For example, Luo et al. [16] reported that the addition of the flavonoid, puerarin, to the diet significantly increased the carcass weight and dressing percentage of beef cattle. Similarly, Wang et al. [17] found that the addition of mulberry flavonoids improved the dressing percentage of rams. In this study, the significant increase in dressing percentage and eye muscle area in the SBS24 group supports our hypothesis that SBS can enhance meat yield. This effect may be attributed to the flavonoids in SBS that promote muscle cell proliferation and muscle tissue synthesis. Additionally, the modulation of gut microbiota by SBS may indirectly affect metabolic pathways and nutrient absorption in rams, leading to better carcass quality. There is evidence that [18] the gut microbiota play a role in protein digestion and absorption, gut barrier function, and inflammation, which are all related to muscle growth and development. However, the decline in slaughter performance indicators in the 30% SBS group indicates that the addition of SBS should be optimized to avoid adverse effects. Possible reasons for this decline could include reduced digestibility, excessive fiber, or anti-nutritional factors present at higher levels of SBS inclusion.

### 4.1 Effects of SBS on rumen fermentation

Rumen pH is a comprehensive indicator of rumen fermentation, reflecting microbial activity and the decomposition status of nutrients, as well as the stability of the rumen environment [19]. The normal range of rumen pH is 5.5–7.5, and deviations from this range can negatively impact rumen fermentation [20]. In this study, the rumen pH values were higher than the typical normal range. The higher pH observed in our study could be a result of the specific fermentation characteristics of SBS, which could alter the production and absorption rates of VFAs in the rumen. Ammonia nitrogen (NH3-N) concentration reflects the degradation of dietary nitrogen and microbial utilization efficiency in the rumen [21–22]. The observed trend of decreased NH3-N concentration with increasing levels of SBS suggests that the active compounds in the straw, such as baicalin, may enhance microbial nitrogen utilization efficiency.

Volatile fatty acids (VFAs) are crucial indicators of rumen fermentation in ruminants, reflecting both the intensity of fermentation and dietary conditions [23–24]. The elevated total VFA concentrations in the SBS groups, particularly in the SBS24 group, suggest enhanced carbohydrate fermentation and VFA synthesis, potentially due to the flavonoid compounds in SBS [25]. Acetate, a key precursor for triglyceride synthesis in ruminants, is closely linked to glucose in fat synthesis [26]. The quadratic change in acetate concentration, with an initial increase followed by a decrease, might be due to the complex interactions between dietary components and rumen microbiota. Butyrate is known to promote the proliferation and differentiation of rumen epithelial cells, thereby increasing the rumen surface area and enhancing its absorption and secretion capabilities, which are vital for energy metabolism [27]. The higher concentrations of acetate and butyrate in the SBS24 group are associated with improved energy utilization efficiency and growth performance in rams, as evidenced by the higher average daily gain observed in this group [28]. The decline in VFA concentrations in the SBS30 group indicates that excessive SBS levels may negatively impact rumen microbiota and fermentation.

### 4.2 Effects of SBS on rumen microbiota

The rumen microbiota is primarily responsible for energy production in ruminants. Studies have shown that differences in the rumen microbial community can alter energy efficiency [29]. Alpha diversity reflects the richness and diversity of species in a sample. The ACE and Chao1 indices are related to microbial community richness, with higher values indicating more species and greater richness. In contrast, the Shannon and Simpson indices are related to microbial community diversity, with higher Simpson indices and lower Shannon indices indicating greater diversity [30]. In this study, the Simpson indices of the SBS groups were significantly higher than that of the CON group. The Shannon indices of the SBS12, SBS18, and SBS24 groups were significantly lower than that of the CON group, and the SBS24 group also had a significantly lower Shannon index than the SBS30 group. This suggests that the % addition of SBS can regulate the abundance and diversity of the rumen microbiota, but excessive levels of SBS can lead to decreased richness and diversity of rumen bacteria.

At the phylum level, the addition of SBS to the diet increased the relative abundance of Bacteroidetes, Firmicutes, and Halobacteria in the rumen, while decreasing the relative abundance of Proteobacteria and Actinobacteria. Bacteroidetes is an important group of bacteria that promotes the degradation of polysaccharides, proteins, and carbohydrates in the diet of ruminants, primarily converting non-fibrous materials into acetate and propionate [31]. This aligns with the higher concentrations of acetate and propionate observed in the SBS groups compared to the CON group. Firmicutes are associated with energy metabolism and can produce various digestive enzymes to break down nutrients, making it a key bacterial group for improving fiber utilization and energy production [32]. The increase in Firmicutes suggests enhanced fiber digestion and energy metabolism in the rumen. Halobacteria have a strong ability to adapt to unfavorable environments [33], and the relative abundance of Halobacteria in the SBS group was increased in this experiment, suggesting that the addition of SBS to the ration may enhance the resistance of the animals. Proteobacteria are mainly Gram-negative and include pathogens such as *Escherichia coli* and *Salmonella*, which can cause diarrhea and inflammation in animals [34]. The decrease in Proteobacteria indicates a potential reduction in harmful bacteria, contributing to improved gut health. Actinobacteria are Gram-positive bacteria, mostly saprophytic, and some are pathogenic [35]. The reduction in Actinobacteria further supports the idea that SBS promotes beneficial microbial balance in the rumen.

The genus *Prevotella* can degrade cellulose, participate in microbial metabolism, hydrolyze proteins and starch, and ferment sugars and amino acids [36]. The genus *Succiniclasticum* has high fiber-degrading activity and can break down crystalline cellulose that white rumen cocci cannot degrade, thereby promoting the digestibility of nutrients in the rumen and increasing the concentration of total volatile fatty acids (TVFAs), which in turn promotes animal growth [37]. In this study, the addition of SBS to the diet showed a trend of increasing TVFA concentration in the rumen, consistent with the microbiota results. The addition of SBS to the diet in this study increased the abundance of the *Rikenellaceae* RC9 gut group, which can effectively degrade soluble polysaccharides and insoluble cellulose, playing an important role in maintaining gut health [38].

The results of this study showed that the addition of 24% SBS increased the relative abundance of *Haloarcula*, which has strong adaptability to adverse environments. Further research is needed to explore its specific function in the rumen. The CON group showed enrichment in some toxic bacteria, such as *Desulfobacter*, which belongs to the phylum Proteobacteria. *Desulfobacter* can affect the pH of the rumen environment, thereby influencing the diversity and richness of the rumen microbial community. The specific mechanisms require further investigation.

The positive correlation between carcass weight and *Succiniclasticum* prevalence (*P* < 0.05) suggests a possible role of this genus in enhancing host growth and fat deposition, which may be linked to its known metabolic functions in succinate utilization and energy metabolism. Similarly, the negative correlation between eye muscle area and *Prevotella* (*P* < 0.05) could indicate that too high an abundance of *Prevotella* might be associated with reduced muscle development, possibly due to its role in fiber degradation and energy partitioning within the host. The negative correlations observed between dressing percentage and propionate with *Prevotellaceae*_UCG_003 (*P* < 0.05) further highlight the complex interplay between microbial communities and host productivity. Propionate, a major energy-yielding VFA in ruminants, is closely tied to host metabolism, and may reflect differences in microbial fermentation efficiency or energy allocation. Additionally, the negative correlation between NH3-N and unclassified_*Prevotellaceae* (*P* < 0.05) suggests a potential regulatory role of this microbial group in nitrogen metabolism, which could influence host protein synthesis and tissue development. Further studies are warranted to elucidate the mechanistic basis of these correlations and to explore their practical application in livestock management.

## 5 Conclusions

Feeding diets containing 6%−30% SBS increased the relative abundance of Firmicutes, Bacteroidetes, and Halobacteria, while decreasing the relative abundance of Proteobacteria and Actinobacteria. This led to higher total VFA, which in turn improved the slaughter performance of rams. SBS at 24% inclusion level improved dressing percentage by 10% and increased butyrate production by 48%. It is recommended that farmers consider incorporating up to 24% SBS in the diets of rams to improve fermentation efficiency and meat yield.

## References

[1] Yang XY, Liu CR, Zhao T, Wu XW, Li M, Duan GJ. Application prospects of Scutellaria baicalensis stems and leaves as feed additives. Specialty Research. 2021;43(4):115–8, 123. doi: 10.16720/j.cnki.tcyj.2021.103

[2] Chen YQ. Application of by-products of traditional Chinese medicine as feed and their effects on growth performance, slaughter performance, and economic benefits in fattening pigs. China Feed. 2023;2023(16):126–9. doi: 10.15906/j.cnki.cn11-2975/s.20231632

[3] Liu XL, Li CY, Chen QJ, Xue JT. Research progress on main active components and pharmacological action of Scutellaria baicalensis. Journal of Xinxiang Medical College. 2023;40(10):979–85. doi: 10.7683/xxyxyxb.2023.10.014

[4] Jiang XH, Liu SM. Study on pharmacological action and chemical substance basis of Scutellaria baicalensis Georgi. China Pharmacist. 2020;23:2004–10. doi: 10.13748/j.cnki.issn1007-7693.2022.19.018

[5] Luo Y Z. Research progress on chemical constituents and pharmacological effects of Scutellaria baicalensis[J]. Journal of Rational Drug Use. 2018;11(30):180–181. doi: 10.1177/1934578x241266692

[6] Gao XH, Zhang JL, Yue CJ, Liang XJ. Effects of stems and leaves of Scutellaria baicalensis on growth performance and serum biochemical, immune, and antioxidant indexes of Simmental crossbred cattle. Chinese Journal of Animal Nutrition. 2022;34(6):3724–31. doi: 10.3969/j.issn.1006-267x.2022.06.033

[7] Zhang MH, Zhang H, Zhang ZJ, Guo TJ. Research progress of bioactive substances from Scutellaria baicalensis straw and application of Scutellaria baicalensis straw and its extract in livestock production. Chinese Journal of Animal Nutrition. 2024;36(9):5557–65.

[8] Liao H, Ye J, Gao L, Liu Y. The main bioactive compounds of Scutellaria baicalensis Georgi. for alleviation of inflammatory cytokines: A comprehensive review. Biomed Pharmacother. 2021;133:110917. doi: 10.1016/j.biopha.2020.110917 33217688

[9] Jiang HL. Study on extraction and microbial transformation of Scutellaria baicalensis roots and stems and leaves. Master’s Thesis[D]. Nanjing: Nanjing Normal University; 2021.

[10] Zhang F, Jin E, Liu X, Ji X, Hu H. Effect of dietary Fructus mume and Scutellaria baicalensis Georgi on the fecal microbiota and its correlation with apparent nutrient digestibility in weaned piglets[J]. Animals. 2022;2022:12. doi: 10.3390/ani12030289 36139277 PMC9495044

[11] Wei QQ, Ma L, Liu XY, Chang ZJ, Yang QL, Zhao M. Effects of compound traditional Chinese medicine additives on rumen fermentation and microbial community structure in sika deer during the antler-growth period. Feed Industry. 2023;44(05):96–100. doi: 10.13302/j.cnki.fi.2023.05.017

[12] Zhang J, Bai X, Na R, Wang X Q, Ma Y, Liang X J. Effect of adding stems and leaves of Scutellaria baicalensis Georgi to feed on the microbial diversity of cattle feces[J]. Research Square. Preprint, (Version 1) Research Square. 2023. doi: 10.21203/rs.3.rs-3241166/v1

[13] Xiao YQ, Zhang YF, Yang RF. Preliminary study on approximate calculation formula for eye muscle area of sheep. China Ruminant Animal. 2003;S1:117–8.

[14] Panche AN, Diwan AD, Chandra SR. Flavonoids: An overview. Journal of Nutritional Science. 2016;5. doi: 10.1017/s1355198X16047846 PMC5465813 28620474

[15] van Zonneveld M, Ramirez M, Williams DE, Petz M, Meckelmann S, Avila T, et al. Screening Genetic Resources of Capsicum Peppers in Their Primary Center of Diversity in Bolivia and Peru. PLoS One. 2015;10(9):e0134663. doi: 10.1371/journal.pone.0134663 26402618 PMC4581705

[16] Luo L N, Li G. Effect of puerarin on growth performance, slaughter performance, and serum antioxidant indexes of beef cattle[J]. Feed Industry. 2024;45(3):56–9. doi: 10.1111/asj.13543 33738872

[17] Wang ZH, He YJ, Wang CL, Zhang ZP, Zhong HM, Li AP. Effects of mulberry flavonoids in feed on growth performance, meat quality, and serum biochemical indicators in meat sheep. China Feed. 2024;16:53–6. doi: 10.15906/j.cnki.cn11-2975/s.20241614

[18] Baky MH, Elshahed M, Wessjohann L, Farag MA. Interactions between dietary flavonoids and the gut microbiome: a comprehensive review. Br J Nutr. 2022;128(4):577–91. doi: 10.1017/S0007114521003627 34511152

[19] Guo CY, Zhang TL, Xu TT, Ao CJ, Wang LF. Effects of compound plant extracts on rumen fermentation of Holstein cows in vitro. Chinese Journal of Animal Nutrition. 2020;32(8):3698–707.

[20] Yang ZM, Shang XL, Chen H, Lan J, Nie CT, Chen H, et al. Chinese Journal of Animal Nutrition. 2022;34(7):4487–97. doi: 10.1186/s40168-020-0064-8

[21] Peng SY. Effects of feeding different levels of wet-stored corn on rumen fermentation, production performance, and rumen flora diversity of dairy cows. Daqing: Heilongjiang Bayi Agricultural University; 2023.

[22] Zhao Y B, Zhang T L, Zhang Y M, Bai C, Ao C J. Effects of cottonseed protein hydrolysate on rumen fermentation paramseters and microbial flora in dairy cows[J]. Chinese Journal of Animal Nutrition. 2021;33(6):3297–3308. doi: 10.3389/fvets.2022.984634 36439362 PMC9698919

[23] Li J, Tuo Y, He L, Ma Y, Zhang Z, Cheng Z, et al. Effects of chili straw on rumen fermentation, meat quality, amino acid and fatty acid contents, and rumen bacteria diversity in sheep. Front Microbiol. 2025;15:1525612. doi: 10.3389/fmicb.2024.1525612 39877758 PMC11773153

[24] Bai QC. Effects of Hippophae rhamnoides flavonoids on growth performance, digestion and metabolism, rumen fermentation, and blood indexes of sheep. Taigu: Shanxi Agricultural University. 2020.

[25] Yang J, Feng F, Li H, Gao Q X. Effects ofC rapeseed as forage on rumen fermentation, nutrient digestion, and meat quality in Tan sheep[J/OL]. Science of Forage and Grassland, 1-26[2025-01-13]. https://kns-cnki-net.webvpn.xjau.edu.cn/kcms/detail/62.1069.S.20250105.1917.008.html

[26] Tan Z X, Bai X, Guo C H, Peng C L. Effects of adding yeast culture to diet on growth performance, slaughter performance, meat quality, and rumen microbial diversity in yaks[J]. Journal of Animal Nutrition. 2024;36(9):5761–75. doi: 10.3389/frmbi.2025.1545689

[27] Sun J. Research progress on the application of butyric acid in ruminants. Jilin Animal Husbandry and Veterinary Medicine. 2024;45(10):139–41. doi: 10.12418/CJAN2023.508

[28] Zhang MH, Guo TJ, Zhang H. Effects of different Scutellaria baicalensis straw levels on growth performance, nutrient apparent digestibility and serum indexes of scape goat. Chinese Journal of Animal Nutrition. 2025;37(4):2546–57. doi: 10.12418/CJAN2025.214

[29] Hanlon ME, Simoni M, Moorby JM, Righi F, Tsiplakou E, Kantas D, et al. Effects of the addition of non-fibre carbohydrates with different rumen degradation rates in dairy cow high-forage diets using the Rumen Simulation Technique. Animal. 2023;17(4):100732. doi: 10.1016/j.animal.2023.100732 36905776

[30] Chen Y, Gu Z, Guo T, Guo X. Effects of different levels of Tagetes erecta meal on rumen bacterial diversity in sheep[J]. Journal of Animal Nutrition. 2023;35(07):4453–64. doi: 10.5455/javar.2020.g477 33409322 PMC7774798

[31] Long Y, Xiao W, Zhao Y, Yuan C, Wang D, Yang Y, et al. Effects of Flammulina velutipes mushroom residues on growth performance, apparent digestibility, serum biochemical indicators, rumen fermentation and microbial of Guizhou black goat. Front Microbiol. 2024;15:1347853. doi: 10.3389/fmicb.2024.1347853 38328420 PMC10848151

[32] Joo R R D, Gouvêa V N, Neto L R D A, Silva S C D, Brink G E, Pires A V, et al. Beef cattle responses to pre-grazing sward height and low level of energy supplementation on tropical pastures[J]. Journal of Animal Science. 2020;98(6):163. doi: 10.2527/jas.2020.0004 PMC7301309 32413898

[33] Xue M-Y, Sun H-Z, Wu X-H, Liu J-X, Guan LL. Multi-omics reveals that the rumen microbiome and its metabolome together with the host metabolome contribute to individualized dairy cow performance. Microbiome. 2020;8(1):64. doi: 10.1186/s40168-020-00819-8 32398126 PMC7218573

[34] Söllinger A, Tøsdal Tveit A, Poulsen M, Noel SJ, Bengtsson M, Bernhardt J. Holistic assessment of rumen microbiome dynamics through quantitative metatranscriptomics reveals multifunctional redundancy during key steps of anaerobic feed degradation. mSystems. 2018;3(4):e00038-18. doi: 10.1128/msys.00075-18 PMC6081794 30116788

[35] Tian R, Ning D, He Z, Zhang P, Spencer SJ, Gao S, et al. Small and mighty: adaptation of superphylum Patescibacteria to groundwater environment drives their genome simplicity. Microbiome. 2020;8(1):51. doi: 10.1186/s40168-020-00825-w 32252814 PMC7137472

[36] Lu X, Xu H, Fang F, Liu J, Wu K, Zhang Y, et al. In vitro effects of two polysaccharide fractions from Laminaria japonica on gut microbiota and metabolome. Food Funct. 2023;14(7):3379–90. doi: 10.1039/d2fo04085a 36943742

[37] Li Q, Tu Y, Ma T, Cui K, Zhang J, Diao Q, et al. Effects of Two Feeding Patterns on Growth Performance, Rumen Fermentation Parameters, and Bacterial Community Composition in Yak Calves. Microorganisms. 2023;11(3):576. doi: 10.3390/microorganisms11030576 36985149 PMC10058967

[38] Zhu Y, Wang Z, Hu R, Wang X, Li F, Zhang X, et al. Comparative study of the bacterial communities throughout the gastrointestinal tract in two beef cattle breeds. Appl Microbiol Biotechnol. 2021;105(1):313–25. doi: 10.1007/s00253-020-11019-7 33201274

